# AT(N) biomarker profiles and Alzheimer's disease symptomology in Down syndrome

**DOI:** 10.1002/alz.13446

**Published:** 2023-08-28

**Authors:** Sigan L. Hartley, Benjamin Handen, Dana Tudorascu, Laisze Lee, Annie Cohen, Emily K. Schworer, Jamie C. Peven, Matthew Zammit, William Klunk, Charles Laymon, Davneet Minhas, Weiquan Luo, Shahid Zaman, Beau Ances, Gregory Preboske, Bradley T. Christian, the Alzheimer Biomarker Consortium – Down Syndrome

**Affiliations:** ^1^ Waisman Center University of Wisconsin–Madison Madison Wisconsin USA; ^2^ School of Human Ecology University of Wisconsin–Madison Madison Wisconsin USA; ^3^ Department of Psychiatry University of Pittsburgh Pittsburgh Pennsylvania USA; ^4^ Department of Medical Physics University of Wisconsin–Madison Madison Wisconsin USA; ^5^ Department of Radiology University of Pittsburgh Pittsburgh Pennsylvania USA; ^6^ Department of Bioengineering University of Pittsburgh Pittsburgh Pennsylvania USA; ^7^ Department of Psychiatry University of Cambridge Cambridge UK; ^8^ Department of Neurology Washington University at St. Louis St. Louis, Missouri USA; ^9^ Mayo Clinic Rochester Minnesota USA

**Keywords:** adults, Alzheimer's, amyloid, ATN, biomarker, cognitive, dementia, Down syndrome, hippocampal, imaging, magnetic resonance imaging, memory, positron emission tomography, tau

## Abstract

**INTRODUCTION:**

Down syndrome (DS) is a genetic cause of early‐onset Alzheimer's disease (AD). The National Institute on Aging–Alzheimer's Association AT(N) Research Framework is a staging model for AD biomarkers but has not been assessed in DS.

**METHOD:**

Data are from the Alzheimer's Biomarker Consortium–Down Syndrome. Positron emission tomography (PET) amyloid beta (Aβ; 15 mCi of [^11^C]Pittsburgh compound B) and tau (10 mCi of [^18^F]AV‐1451) were used to classify amyloid (A) –/+ and tau (T) +/–. Hippocampal volume classified neurodegeneration (N) –/+. The modified Cued Recall Test assessed episodic memory.

**RESULTS:**

Analyses included 162 adults with DS (aged M = 38.84 years, standard deviation = 8.41). Overall, 69.8% of participants were classified as A–/T–/(N)–, 11.1% were A+/T–/(N)–, 5.6% were A+/T+/(N)–, and 9.3% were A+/T+/(N)+. Participants deemed cognitively stable were most likely to be A–T–(N)– and A+T–(N)–. Tau PET (T+) most closely aligning with memory impairment and AD clinical status.

**DISCUSSION:**

Findings add to understanding of AT(N) biomarker profiles in DS.

**Highlights:**

- Overall, 69.8% of adults with Down syndrome (DS) aged 25 to 61 years were classified as amyloid (A)–/tau (T)–/neurodegeneration (N)–, 11.1% were A+/T–/(N)–, 5.6% were A+/T+/(N)–, and 9.3% were A+/T+/(N)+.
- The AT(N) profiles were associated with clinical Alzheimer's disease (AD) status and with memory performance, with the presence of T+ aligned with AD clinical symptomology.
- Findings inform models for predicting the transition to the prodromal stage of AD in DS.

## 1 BACKGROUND

People with Down syndrome (DS), or trisomy 21, are genetically at risk for Alzheimer's disease (AD).^1^ The triplication of the amyloid precursor protein gene located on chromosome 21 results in an overproduction of amyloid beta (Aβ),^2^ which aggregates into extracellular Aβ plaques in adulthood and is associated with a cascade of other pathologic effects that are thought to cause AD.^3^, ^4^ The median age of AD dementia onset in DS is 53.8 years^5^ and there is a 90% lifetime incidence.^6^, ^7^

The hypothetical model of AD in DS^8^, ^9^, ^10^ is thought to be similar to autosomal dominant AD (i.e., resulting from mutations in amyloid precursor protein, presenilin 1 or 2 genes) and is based primarily on cross‐sectional findings that draw on a range of biomarkers—cerebrospinal fluid, plasma, and neuroimaging. In terms of neuroimaging, positron emission tomography (PET) biomarkers of extracellular Aβ plaques occur by the fourth and fifth decade of life^11^, ^12^ and intracellular neurofibrillary tangles of tau in the fifth decade in DS.^13^, ^14^ Studies have also reported magnetic resonance imaging (MRI)‐based hippocampal volume decline^15^, ^16^ by the fifth decade of life in DS. Outside of DS, AD is characterized by a lengthy preclinical stage in which AD pathology increases prior to the onset of clinical dementia.^17^, ^18^, ^19^ Research to date suggests that this prolonged preclinical stage is also true in DS, with the above brain changes documented prior to onset of clinical dementia.^1^, ^11^, ^20^, ^21^, ^22^, ^23^

During this preclinical stage, biomarkers of AD pathology including those of Aβ, tau, and neurodegeneration are thought to have prognostic value with regard to understanding which individuals are likely to develop AD dementia in the coming years. The National Institute on Aging–Alzheimer's Association Research Framework was developed to provide a staging model referred to as the AT(N).^18^, ^19^ This framework classes individuals into groups based on three types of biomarkers: amyloid (A), tau (T), and neurodegeneration (N), which are theorized to drive symptomatic AD.^19^

Within the AT(N) framework, there are eight possible classifications that range and rank from all biomarkers being negative (A−T−(N)−) to all positive (A+T+(N)+). Based on the theoretical model of AD proposed by Jack et al.,^18^, ^19^ the presence of A+ usually precedes T+, which is typically followed by (N)+ or neurodegeneration that is often indexed by structural MRI biomarkers such as hippocampal atrophy. Within this classification system, the A–T–(N)– group would be expected to be furthest from clinical dementia onset and the A+T+(N)+ group closest.^24^, ^25^, ^26^ A growing body of work has reported on individual biomarkers and their link to cognitive performance and clinical dementia in DS.^8^, ^15^, ^16^, ^17^, ^20^, ^21^, ^22^, ^23^ However, DS research has yet to combine AT(N) biomarkers using the AT(N) classification system.

The study aims were to: (1) examine AT(N) profiles in DS based on PET Aβ (A), tau PET (T), and MRI hippocampal volume ([N]) and (2) evaluate potential differences in clinical AD status and memory by AT(N) group. Based on the Jack et al.^19^ hypothesized AD pathological processes and research findings on individual biomarkers in DS, it was hypothesized that the presence of A+ would precede T+, which would precede (N)+, such that T+ in the absence of A+ would be rare, as would (N)+ in the absence of A+ and T+. The A+T+(N)+ group was predicted to have a higher prevalence of mild cognitive impairment (MCI) or dementia and be associated with poorer memory performance than the other groups. The A–T–(N)– group was predicted to have the lowest prevalence of MCI or dementia and be associated with the highest memory performance of all the groups, with the A+T–(N)– and A+T+(N)– groups in the middle.

## 2 METHODS

### 2.1 Participants

Participants were 162 adults with DS, with a mean age of 38.84 (standard deviation [SD] = 8.41) who completed a baseline assessment at one of five sites (University of Wisconsin, University of Pittsburgh, Washington University–St. Louis, University of Cambridge, and Barrow Neurological Institute) in the Alzheimer's Biomarker Consortium–Down Syndrome. Participants were required to be ≥ 25 years, have genetic confirmation of DS, and have at least minimal verbal communication skills. Participants were excluded if they had an unstable medical condition that altered cognition or a condition that did not allow for brain imaging. Informed consent was obtained prior to participation and only de‐identified data was shared across sites. Table 1 includes participant sociodemographics.

**TABLE 1:** Sociodemographics, memory, clinical status, and biomarker variables by AT(N) classification.

	Total (*N* = 162)	A–T–(N)–(*N* = 113)	A+T–(N)–(*N* = 18)	A+T+(N)–(*N* = 9)	A+T+(N)+(*N* = 15)	A–T–(N)+(*N* = 2)	A–T+(N)–(*N* = 4)	A+T–(N)+(*N* = 1)
Age in years (M, SD)	38.84 (8.51)	34.92 (5.93)	46.57 (5.07)	51.50 (4.02)	50.79 (5.15)	42.87 (8.92)	35.63 (2.72)	53.56 (–)
Female (*N*, %)	77 (46%)	54 (48%)	10 (56%)	1 (11%)	7 (47%)	1 (50%)	3 (75%)	1 (100%)
Intellectual disability (*N*,%)								
Mild	61 (38%)	45 (40%)	5 (28%)	4 (44%)	5 (33%)	1 (50%)	1 (25%)	0 (0%)
Moderate	95 (59%)	63 (65%)	13 (72%)	5 (56%)	9 (60%)	1 (50%)	3 (75%)	1 (100%)
Severe/profound	6 (4%)	5 (3%)	0 (0%)	0 (0%)	1 (7%)	0 (0%)	0 (0%)	0 (0%)
mCRT Total (M,SD)	31.35 (6.80)	32.83 (4.83)	31.82 (8.00)	24.74 (7.38)	20.46 (8.72)	33.50 (2.12)	35.50 (1.00)	36.00 (–)
mCRT Intrusions (M,SD)	3.36 (4.70)	2.20 (2.70)	2.65 (2.62)	9.25 (7.27)	11.69 (7.79)	2.50 (2.12)	0.50 (1.00)	3.36 (4.70)
Clinical status (*N*,%)								
Cognitively stable	140 (86%)	108 (96%)	16 (89%)	4 (44%)	5 (33%)	2 (100%)	4 (100%)	1 (100%)
MCI	7 (4%)	2 (2%)	0 (0%)	2 (22%)	3 (20%)	0 (0%)	0 (0%)	0 (0%)
Dementia	6 (4%)	1 (1%)	0 (0%)	1 (1%)	4 (27%)	0 (0%)	0 (0%)	0 (0%)
Unable to determine	9 (6%)	2 (2%)	2 (11%)	2 (22%)	3 (20%)	0 (0%)	0 (0%)	0 (0%)
PET Aβ (M,SD)	16.65 (28.19)	2.18 (5.19)	31.41 (10.43)	65.78 (18.58)	78.43 (24.18)	0.00 (0.00)	−1.93 (2.43)	30.88 (–)
Tau PET (M,SD)	1.20 (0.25)	1.11 (0.06)	1.13 (0.05)	1.51 (0.27)	1.79 (0.31)	1.02 (0.01)	1.24 (0.03)	1.04 (–)
Hippocampal vol. (M, SD)	6960.76 (1022.97)	7260 (812.11)	6753.93 (555.82)	6740.79 (785.93)	5084.35 (961.28)	4071.25 (2684.39)	7338.65 (435.31)	5221.80 (–)
Intracranial vol. (M, SD)	1252863.20 (176135.49)	1240091.24 (171047.53)	1205527.48 (208904.41)	1392608.77 (149959.57)	1322034.13 (139576.93)	1020172.48 (541345.77)	1240284.96 (171884.70)	1136484.30 (–)

Abbreviations: A, amyloid; Aβ, amyloid beta in Centiloids; MCI, mild cognitive impairment; mCRT, modified Cued Recall Test; (N), neurodegeneration; PET, positron emission tomography; SD, standard deviation; T, tau; vol., volume.

### 2.2 Measures

#### 2.2.1 Control variables

Age was calculated in years. The participant's premorbid intellectual functioning was assessed using the Stanford‐Binet, fifth edition (SB5^27^) abbreviated IQ subtests prior to a clinical AD status of MCI or dementia. The mental age equivalent scores corresponded to the following intellectual disability levels: mild: ≥ 9 years, moderate: 5 to 8 years, and severe/profound: ≤ 4 years. Study site was arbitrarily coded as University of Pittsburgh = 1, University of Wisconsin–Madison, = 2, Cambridge University = 3, Barrow Neurological Institute = 4, and Washington University–St. Louis = 5 to examine and control for any site differences in models.

#### 2.2.2 Clinical AD status

A case consensus conference determined clinical AD status. This process involved a psychologist, physician, and at least two additional staff experienced in AD in DS. All members were blind to neuroimaging and biofluid data and all available points of data collection were considered for participants enrolled in our legacy studies. Caregiver‐reported information and directly administered measures of dementia symptoms, cognitive functioning, and adaptive behavior were reviewed and considered in the context of the participant's premorbid intellectual disability level, psychiatric and medical conditions, and recent major life events (details on specific measures reported in Handen et al.^28^). This process was based on an overall clinical impression after reviewing the above information, rather than focusing on any one measure or cut‐off score. Team members were provided with published measure norms broken down by premorbid intellectual disability level. Clinical AD statuses included: (1) cognitively stable, meaning no evidence of cognitive or functional decline; (2) MCI, indicating cognitive and/or functional declines that were limited in severity or domain; (3) dementia, indicating cognitive decline of marked severity and decreases in daily functioning; and (4) unable to determine.

**RESEARCH IN CONTEXT**

1. **Systematic review**: Literature was reviewed using traditional (PubMed) sources. While the amyloid/tau/neurodegeneration (AT[N]) framework has been studied in sporadic late onset Alzheimer's disease (AD) and autosomal dominant AD, it has yet to be studied in Down syndrome (DS). This work has been appropriately cited.
2. **Interpretation**: Based on the literature review, we hypothesized the AT(N) staging model would align with the sequence of AD biomarker progression and symptomology in DS.
3. **Future directions**: The results indicated that the AT(N) profiles were related to AD symptomology in DS in similar ways as in sporadic onset and autosomal dominant groups. Thus, the AT(N) staging model may be useful for guiding clinical AD trials in DS. Within‐person change in AT(N) profiles across time and investigation of alternative biomarkers of (N) are needed to advance this area of work.

#### 2.2.3 Memory

The modified Cued Recall Test (mCRT^23^) was used to assess episodic memory. Participants were shown three cards, each with four pictures of objects during the learning trials. During the free recall trials, participants freely recalled as many pictures as possible. In the cued recall trial, a category cue (e.g., “fruit” for picture of grapes) was given for pictures not freely recalled. The Free and Cued Recall scores were summed to create the mCRT Total.^29^ Intrusions (incorrect responses of objects not shown) were summed to create the Intrusion score. The mCRT has been found to be positively associated with Aβ PET and tau PET prior to clinical AD dementia in DS.^21^, ^22^ The mCRT Total score was also shown to distinguish adults with DS with versus without AD dementia.^30^

#### 2.2.4 Magnetic resonance imaging

MRI scans were conducted on 3.0 Tesla scanners: GE Discovery MR750 (Wisconsin, Barrow), Siemens Prisma (Pittsburgh), GE Signa PET/MR (Cambridge), and Siemens Prisma (Washington–St. Louis). High‐resolution T1‐weighted images were acquired using a 3D fast spoiled gradient echo sequence or magnetization prepared rapid‐acquisition gradient echo sequence consistent with the Alzheimer's Disease Neuroimaging Initiative (ADNI) 3 and Human Connectome Project protocols. Images were processed at the Mayo Clinic Aging Imaging Lab with FreeSurfer 5.3^31^ and Desikan–Killiany (DK) atlas.^32^

#### 2.2.5 Hippocampal volume

Hippocampal volume was parsed into left and right volumes (in mm^3^), and then summed. The mean total hippocampal volume for the cognitively stable AD clinical status group was used to create neurodegeneration (N) groupings. Specifically, (N)+ was defined as > 1.5 SD below the mean for participants with DS in the cognitively stable group when controlling for intracranial volume. This approach is in line with previous methods used outside of DS for establishing hippocampal atrophy thresholds when examining AD biomarkers.^33^

#### 2.2.6 PET

PET data were acquired using [^11^C] Pittsburgh compound B (PiB) and [^18^F] AV‐1451 for Aβ and tau quantification, respectively. Tracers were administered as 20 to 30 s bolus injections and saline flush. Images were obtained post‐injection at 50 to 70 minutes for [^11^C] PiB and 80 to 100 minutes for [^18^F] AV‐1451. An iterative method was used for data reconstructing. Data was corrected for deadtime, attenuation, scatter, radioactive decay, and motion using a frame‐by‐frame process, with images acquired in 5‐minute frames.

#### 2.2.7 [11C] PiB and [18F] PET processing

The Centiloid method^34^ was used to quantify [^11^C] PiB PET data via SPM8 software. Images were registered to corresponding T1 MR. Each T1 MR scan was deformed to match the 152‐subject template of the Montreal Neurological Institute [MN152] included with SPM8 and the corresponding PET images were co‐warped using the determined parameters. PiB radioactivity concentration was extracted for the Centiloid standard global region and whole cerebellum. Global standardized uptake value ratio (SUVR) was the ratio of tracer concentration in the global region to that of whole cerebellum. This tissue ratio was converted to Centiloids using the linear+constant transformation specified for [^11^C] PiB.^34^

The 80 to 100 minute [^18^F] AV‐1451 tau images were registered to T1 MRI to determine regions of interest for PET quantitation. PET images were sampled based on FreeSurfer 5.3 parcellation. However, due to anatomical differences in DS versus neurotypical populations used in FreeSurfer atlases and because of motion issues, a multi‐template method was used. T1 MR scans were processed through FreeSurfer 5.3 resulting in each being parcellated into regions defined by the DK atlas.^32^ Results were inspected, and 12 high‐quality scans were selected as templates. FreeSurfer parcellations were edited to better conformation the FreeSurfer–based atlas with the template anatomy.

The 12 templates were warped to the T1 MR using the Advanced Neuroimaging Tools (ANTs) software package^35^, ^36^ resulting in 12 versions of the DK atlas in subject space. Each subject‐space voxel was labeled with the DK region most often chosen by the 12 atlases. All results were accepted or rejected based on a visual rating of the final atlas adherence to subject MR anatomy. In a few cases, the direct application of FreeSurfer, along with editing, produced acceptable parcellations.

The tissue uptake of [^18^F] AV‐1451 was expressed as SUVR and was determined using volume weighted average of tracer concentration within FreeSurfer–based components reproducing the composite region defined by Jack et al.^37^ divided by the cerebellar cortex concentration. The Aβ centiloid value and tau Mayo‐composite SUVR were used to classify participants as A –/+ (threshold value ≥ 18) and T +/– (threshold value ≥ 1.21).^37^ These thresholds were established based on prior cross‐sectional and longitudinal work.^22^, ^38^

### 2.3 Data analysis plan

Histograms and boxplots examined the distribution of variables and screened for outliers. Pearson correlations examined the correlation between biomarkers of A, T, and (N) as continuous variables. Descriptive statistics were used to evaluate number and prevalence of each AT(N) profile using the thresholds for – versus +. A chi‐square test examined the prevalence of AT(N) profile groups by clinical AD status. General linear models controlling for age, site, and premorbid intellectual disability level compared memory performance by AT(N) profile. Model assumptions were checked using residual plots.

## 3 RESULTS

**FIGURE 1:**
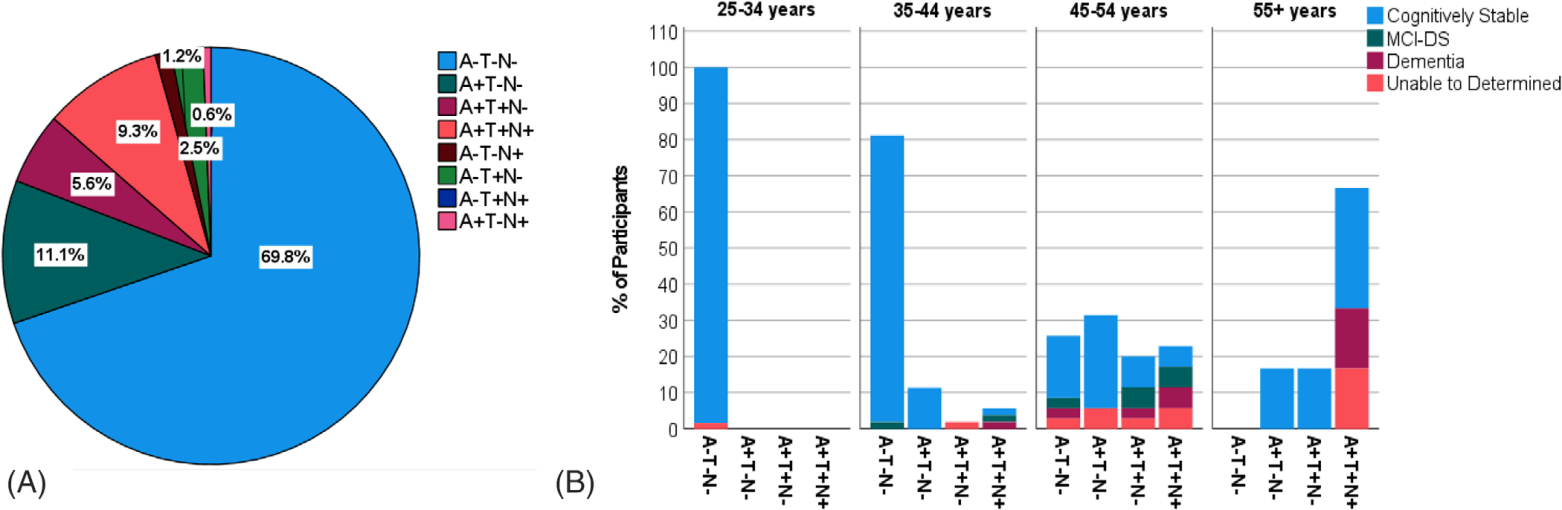
Percentage of the sample by AT(N) profile (A) and percentage of participants in each age group by AT(N) profile and clinical status group (B). A, amyloid; DS, Down syndrome; MCI, mild cognitive impairment; (N), neurodegeneration; T, tau.

Table 1 displays the means and SDs for the AT(N) biomarkers and mCRT. Variables had a normal distribution without skew. There was a significant positive association between A (i.e., PET Aβ) and T (i.e., tau PET) when examined as continuous variables (*r* = 0.824, *P* < 0.001; Figure S1 in supporting information). There was a significant negative association between A and T and (N) (i.e., MRI hippocampal volume adjusted for intracranial volume; A with [N]: *r* = –0.624, *P* < 0.001; T with [N]: *r* = –0.534, *P* < 0.001). Figure 1A displays the percentage of the 162 participants by AT(N) profile using the –/+ thresholds for AT(N) groupings. Overall, 113 (69.8%) participants were A–T–(N)–, 18 (11.1%) were A+T–(N)–, 9 (5.6%) were A+T+(N)–, 15 (9.3%) were A+T+(N)+, 2 (1.2%) were A–T+(N)–, 4 (2.5%) were A+T–(N)+, 1 (0.6%) was A–T–(N)+, and 0 (0%) were A–T+(N)+. Given the small number of participants (7, 4.3%) in the four groups not theorized to be associated with AD (A–T+[N]–, A–T–[N]+, A+T–[N]+, and A–T+[N]+), these groups were not included in remaining analyses.

Figure 1B displays the percentage of participants by age group in each AT(N) profile and clinical AD status group (i.e., cognitively stable, MCI, dementia, and unable to determine). Figure S2 in supporting information displays a scatterplot with loess fit lines for the association between age and Aβ PET and tau PET by clinical status. A chi‐square test indicated a significant difference in AT(N) group by clinical AD status (χ^2^ = 62.874, *P* < 0.001). The percentage of participants who were cognitively stable was higher in the A–T–(N)– and A+T–(N)– groups (95.5% and 88.9%, respectively) than the A+T+(N)– and A+T+(N)+ groups (44.4% and 33.3%, respectively). In contrast, the A–T–(N)– and A+T–(N)– groups had a lower percentage of participants with MCI (1.8% and 0%, respectively) than the A+T+(N)– and A+T+(N)+ groups (22.2% and 20.0%, respectively). The A+T+(N)– and A+T+(N)+ groups had a higher percentage of participants with a dementia status (11.1% and 26.7%) than the A–T–(N)– and A+T–(N)– groups (0.9% and 0%, respectively). The A+T+(N)– and A+T+(N)+ groups also had a higher percentage of participants with unable to determine status (22.2% and 20.0%, respectively) than the A–T–(N)– or A+T–(N)– groups (1.8% and 11.1%, respectively).

**FIGURE 2:**
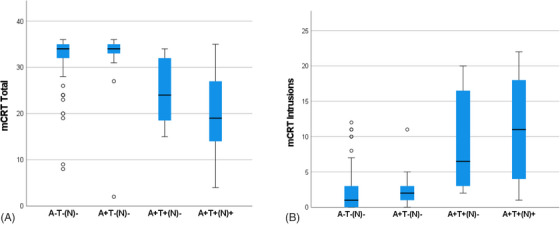
Boxplots of modified Cued Recall Test (mCRT) Total (A) and Intrusions (B) by AT(N) profile group. A, amyloid; (N), neurodegeneration; T, tau.

**FIGURE 3:**
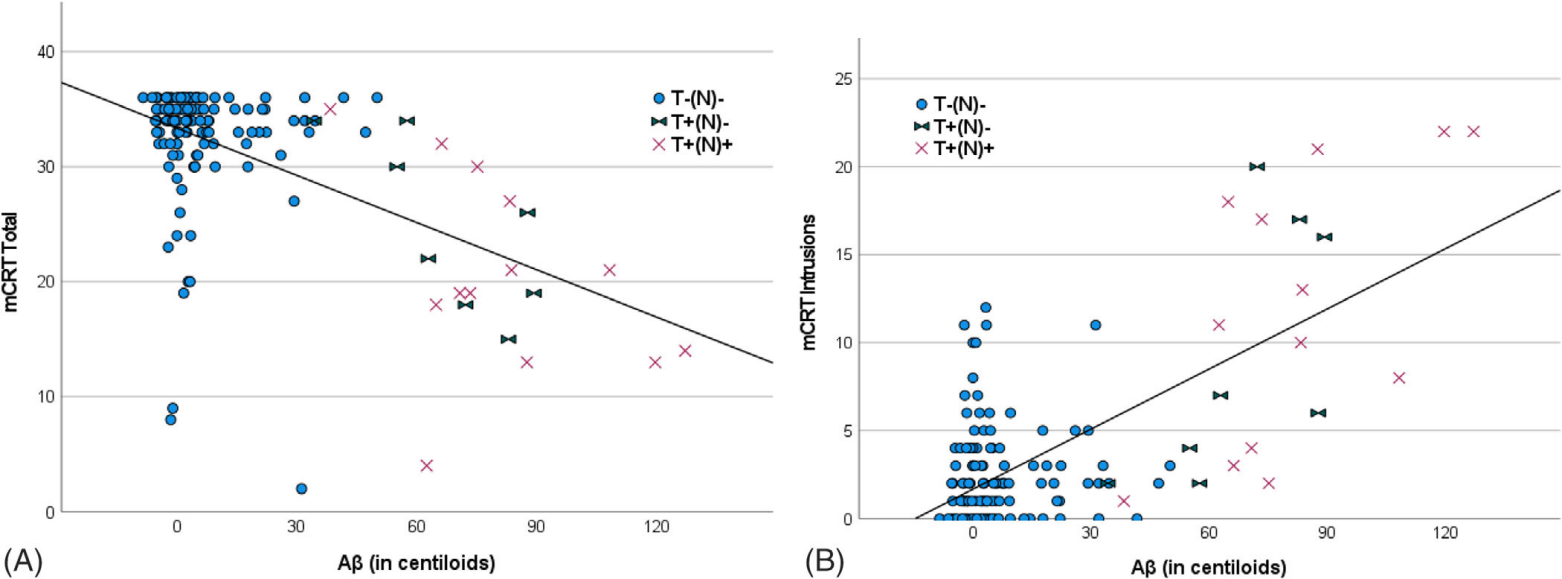
Association between PET Aβ and the modified Cued Recall Test (mCRT) Total (A) and Intrusions (B) scores by tau PET (T) and neurodegeneration ([N]) status. A, amyloid; Aβ, amyloid beta; (N), neurodegeneration; PET, positron emission tomography; T, tau.

Figure S3 in supporting information displays a scatterplot with loess fit lines for the association between age and mCRT Total and Intrusion scores by clinical status. General linear models predicting memory performance (mCRT Total and Intrusion), and controlling for age, site, and premorbid intellectual disability level, indicated significant differences by AT(N) group (Table 2). There was not a significance difference between the A–T–(N)– and A+T–(N)– groups on the mCRT Total (1.197, standard error [SE] = 1.886, *P* = 0.527) or Intrusion (−0.341, SE = 1.228, *P* = 0.782) scores. In contrast, the A+T+(N)– group had significantly lower mCRT Total (−8.200, SE = 2.536, *P* = 0.002 and −7.003, SE = 2.553, *P* = 0.007) and Intrusions (6.745, SE = 1.652, *P* = 0.017 and 6.404, SE = 1.663, *P* = 0.000, respectively) scores than the A–T–(N)– and A+T–(N)– groups. There was not a significance difference between the A+T+(N)– and A+T+(N)+ groups on the mCRT Total (−3.186, SE = 2.586, *P* = 0.220) or Intrusions (1.589, SE = 1.684, *P* = 0.347) scores. Figure 2 displays boxplots of the mean mCRT Total and Intrusion scores by AT(N) group. Figure 3 shows scatterplots of the association between PET Aβ and mCRT performance based on T and (N) –/+ status.

**TABLE 2:** General linear model results for analyses examining effect of AT(N) classification group on memory.

Memory outcome	Variable	Estimate, SE	*P* value	F value, *P* value
mCRT Total	Site (vs. site 1)			4.469, 0.002
	Site 2	−0.505, 1.179	0.669	
	Site 3	−8.273, 2.993	0.007	
	Site 4	−0.996, 1.295	0.433	
	Site 5	−6.840, 2.070	0.001	
	Age	−0.119, 0.086	0.170	
	Intellectual disability level (vs. mild)			2.095, 0.127
	Moderate	−1.845, 0.983	0.063	
	Severe/profound	−2.221, 2.540	0.384	
	AT(N) group			10.782, 0.000
	A–T–(N)– vs. A+T–(N)–	1.197, 1.886	0.527	
	A–T–(N)– vs. A+T+(N)–	−7.003, 2.553	0.007	
	A–T–(N)– vs. A+T+(N)+	−10.190, 2.199	0.000	
	A+T–(N)– vs. A+T+(N)–	−8.200, 2.536	0.002	
	A+T–(N)– vs. A+T+(N)+	−11.387, 2.212	0.000	
	A+T+(N)– vs. A+T+(N)+	−3.186, 2.586	0.220	
mCRT Intrusions	Site (vs. site 1)			2.986, 0.021
	Site 2	0.577, 0.768	0.454	
	Site 3	2.548, 1.950	0.194	
	Site 4	0.911, 0.844	0.282	
	Site 5	4.428, 1.348	0.001	
	Age	−0.002, 0.054	0.955	
	Intellectual disability level (vs. mild)			0.370, 0.691
	Moderate	0.111, 0.671	0.869	
	Severe/Profound	1.494, 1.736	0.391	
	AT(N) Group			15.219, 0.000
	A–T–(N)– vs. A+T–(N)–	−0.341, 1.228	0.782	
	A–T–(N)– vs. A+T+(N)–	6.404, 1.663	0.000	
	A–T–(N)– vs. A+T+(N)+	7.993, 1.433	0.000	
	A+T–(N)– vs. A+T+(N)–	6.745, 1.652	0.017	
	A+T–(N)– vs. A+T+(N)+	8.334, 1.447	0.000	
	A+T+(N)– vs. A+T+(N)+	1.589, 1.684	0.347	

Abbreviations: A, amyloid; mCRT, modified Cued Recall Test; (N), neurodegeneration; SE, standard error; Site 1, University of Pittsburgh; Site 2, University of Wisconsin–Madison; Site 3, Barrow Neurological Institute; Site 4, University of Cambridge; Site 5, Washington University–St. Louis; T, tau.

## 4 DISCUSSION

As the DS research community prepares for clinical AD trials, there is an urgent need to describe the progression of biomarkers of AD pathology and their links with AD symptomology. The present study examined the AT(N) classification system,^18^, ^24^ which was developed outside of DS and is based on a hypothetical model of AD, in a large cohort of adults with DS. The study also determined how the AT(N) classification groups relate to AD symptomology in DS. Findings show marked similarities between AT(N) groups in DS and those reported in autosomal dominant and sporadic late onset AD.^39^, ^40^, ^41^

The breakdown of AT(N) groups was consistent with the hypothetical model of AD in DS.^1^, ^9^, ^10^ The most prevalent groups were A–T–(N)–, A+T–(N)–, A+T+(N)–, and A+T+(N)+, aligning with the proposed time course of Aβ deposition, followed by tau accumulation, and later neurodegeneration in the form of hippocampal atrophy. Classification groups outside of the theorized model of AD pathological progression (A+T–[N]+, A–T–[N]+, and A–T+[N]–) occurred but were rare (*n* = 7, 4%). These groups are also reported in non‐DS populations, including the ADNI sample, at a similar percentage compared to that in this DS sample.^39^, ^40^, ^41^, ^42^ Often, these classification groups are considered to reflect non‐AD processes, but also may reflect spurious false positive or false negatives. The latter may be especially true in this study as all seven of the non‐theorized groupings occurred in participants deemed to be cognitively stable.

The AT(N) classification groups were clearly related to differences in AD symptomology in DS. Groups with elevated PET Aβ and tau (A+T+) were associated with increased likelihood of clinical status of MCI and dementia and poorer memory performance relative to groups without elevated levels of these brain proteins. However, elevated Aβ without elevated tau (A+T–) was not associated with greater AD symptomology in regard to clinical status or memory performance. These findings align with work in sporadic late onset and autosomal dominant AD showing that the presence of T+ often corresponds in time with AD symptomology.^40^, ^41^, ^42^, ^43^, ^44^, ^45^ For example, in a large (*N* = 1431) study involving eight cohorts recruited from memory clinics, clinical trials, and cohort studies, tau PET was a stronger predictor of cognitive change over time than Aβ PET and MRI‐based markers of hippocampal volume and cortical thickness.^44^ Tau PET has also been found to differentiate symptomatic from presymptomatic individuals enrolled in autosomal dominant AD cohorts.^45^

In the present study, the absence (A+T+[N]–) versus presence (A+T+[N]+) of hippocampal atrophy was not associated with differences in AD symptomology for those with elevated PET Aβ and tau PET. It is possible that hippocampal atrophy is not a meaningful indicator of further neurodegeneration progression in AD symptomology beyond the presence of elevated tau PET in DS. Rather, other markers of neurodegeneration may be more useful to reflect symptom progression in DS. Alternatively, the small number of participants in the A+T+N+ group (*N* = 8; 6%) may have obscured effects as there was a trend in the expected direction, particularly in the dementia group.

The unable to determine clinical status cases largely paralleled the MCI and dementia clinical status groups. This suggests that during the transition to prodromal AD there may often have been an uneven range of clinical presentations in individuals with DS, leading to diagnostic uncertainty. While all the individuals with DS in the unable to determine group evidenced some level of cognitive or functional decline, this was only on a subset of measures and/or there was a sporadic profile. Individuals in the unable to determine group often experienced significant life events (e.g., change in residence) that could have accounted for sporadic decline. The unable to determine cases also often involved informant‐reported behavioral and/or mood changes (e.g., irritability or depressed or anxious mood), which added uncertainty about whether cognitive or functional problems were due to emerging AD symptomology or life events. Future longitudinal research is needed to better understand the potential for mood and behavioral changes to be part of early AD symptomology in DS, given previous reports of such changes prior to memory decline similar to frontotemporal dementias.^46^, ^47^, ^48^, ^49^ Longitudinal research will also assist in determining if the unable to determine cases are labeled MCI or dementia at subsequent study visits, as suggested by their AT(N) profile.

The study should be interpreted in light of its both its strengths and limitations. Strengths of the study include the large size of the DS cohort and use of well‐established PET and MRI biomarkers. Moreover, clinical status was based on a rigorous case consensus process and memory performance was directly assessed using an established measure. There were also study limitations, including the modest number of adults with DS in the AT(N) classification groups theorized to be closest to AD dementia (i.e., A+T+[N]+). In addition, thresholds for determining positivity (+) in AT(N) biomarkers including PET Aβ, tau PET, and hippocampal atrophy by structural MRI have yet to be established in DS. The present study drew on thresholds informed by methods used in autosomal dominant AD samples based on deviations from healthy controls and prior research in DS. These thresholds should be investigated in new samples. Other modalities for AT(N) biomarkers, such as plasma and cerebrospinal fluid, should also be examined and compared to the imaging biomarkers. While tau PET appears to have high clinical utility for predicting AD symptomology among imaging biomarkers, it is possible that biomarkers of A and (N) are stronger in plasma or cerebrospinal fluid modalities. Finally, the study was cross‐sectional; future longitudinal analyses are needed to determine whether AT(N) classification groups predict impending onset to AD dementia in DS samples.

In conclusion, this study adds to understanding of AT(N) biomarker profiles in DS and their association with AD symptomology. Results indicate that AT(N) biomarker progression in DS is similar to that observed in sporadic late onset and autosomal dominant AD. These findings can be used to inform models for predicting the transition to the prodromal stage of AD in DS, with the presence of elevated tau PET most closely aligning with clinical AD symptomology. In the coming years, there will be increasing opportunities for AD clinical trials within the DS population. In late onset sporadic AD and autosomal dominant AD populations, AT(N) biomarker status is often part of enrollment criteria to allow for targeted interventions with individuals at relevant stages of disease progression. AT(N) biomarkers can play a similar role in enrollment into AD clinical trials for the DS population, with tau PET being a prognostic marker of AD symptomology.

## CONFLICT OF INTEREST STATEMENT

GE Healthcare holds a license agreement with the University of Pittsburgh for the [11C]PiB PET technology involved in this study. William Klunk is a co‐inventor of [11C]PiB and has financial interest in this license agreement. GE Healthcare did not provide financial support for this study nor did it have a role in designing the study or interpreting the results. None of the other authors have conflicts of interest to disclose. Author disclosures are available in the supporting information.

## CONSENT STATEMENT

All human subjects provided informed consent and/or assent with consent provided by their legal guardian.

## Supporting information

Supporting information

Supporting information
