# Supplementary material for: AT(N) biomarker profiles and Alzheimer's disease symptomology in Down syndrome
Source: Alzheimers Dement. 2023 Aug 28;20(1):366–75. doi: 10.1002/alz.13446 (PMC10840615; doi:10.1002/alz.13446)
Supplement: Supplementary file 1 — Supporting information [file ALZ-20-366-s002.pdf]

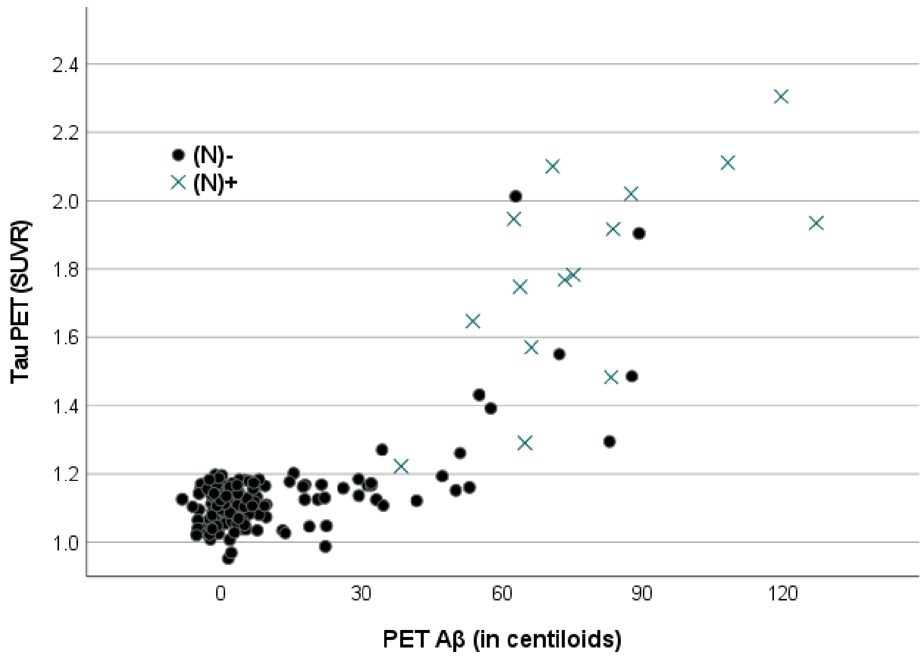

Supplemental Figure e1. Association between PET Aβ and tau PET in standardized uptake value ratio (SUVR) by neurodegeneration ([N] – vs. [N]+) status.

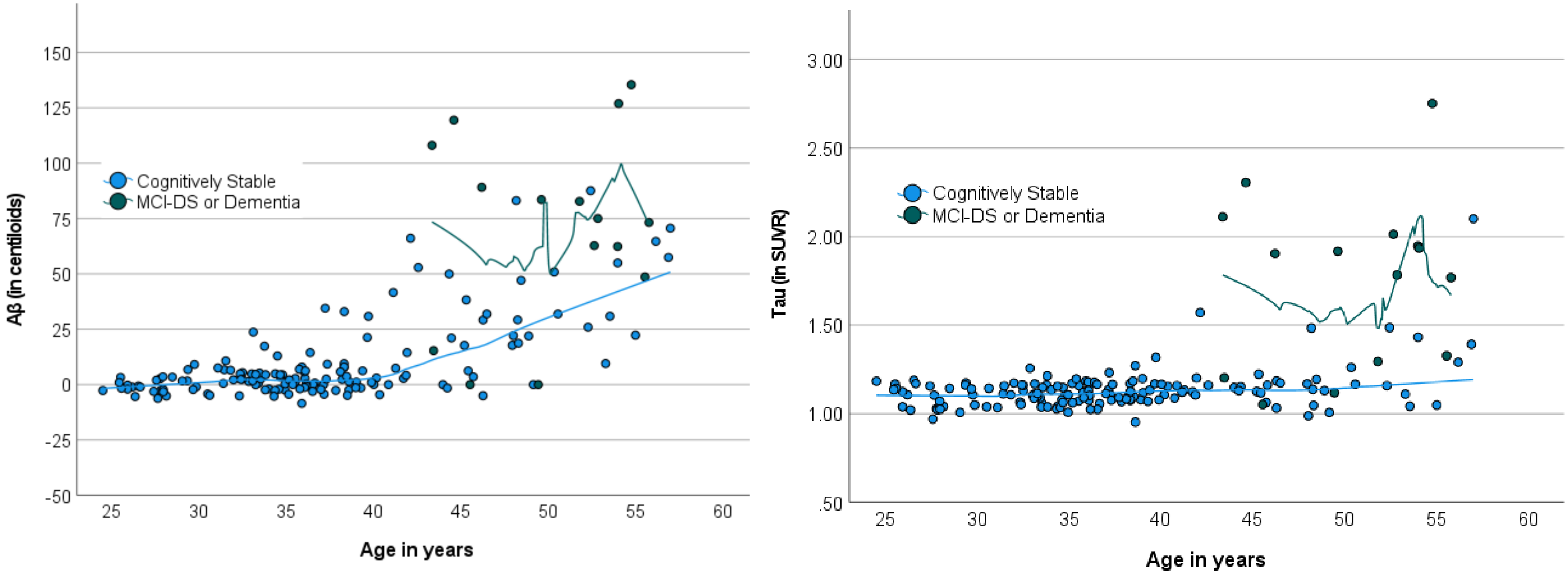

Supplemental Figure e2. Association between age in years and PET Aβ (A) and tau PET (B) by clinical status of mild cognitive impairment-Down syndrome (MCI-DS) and dementia.

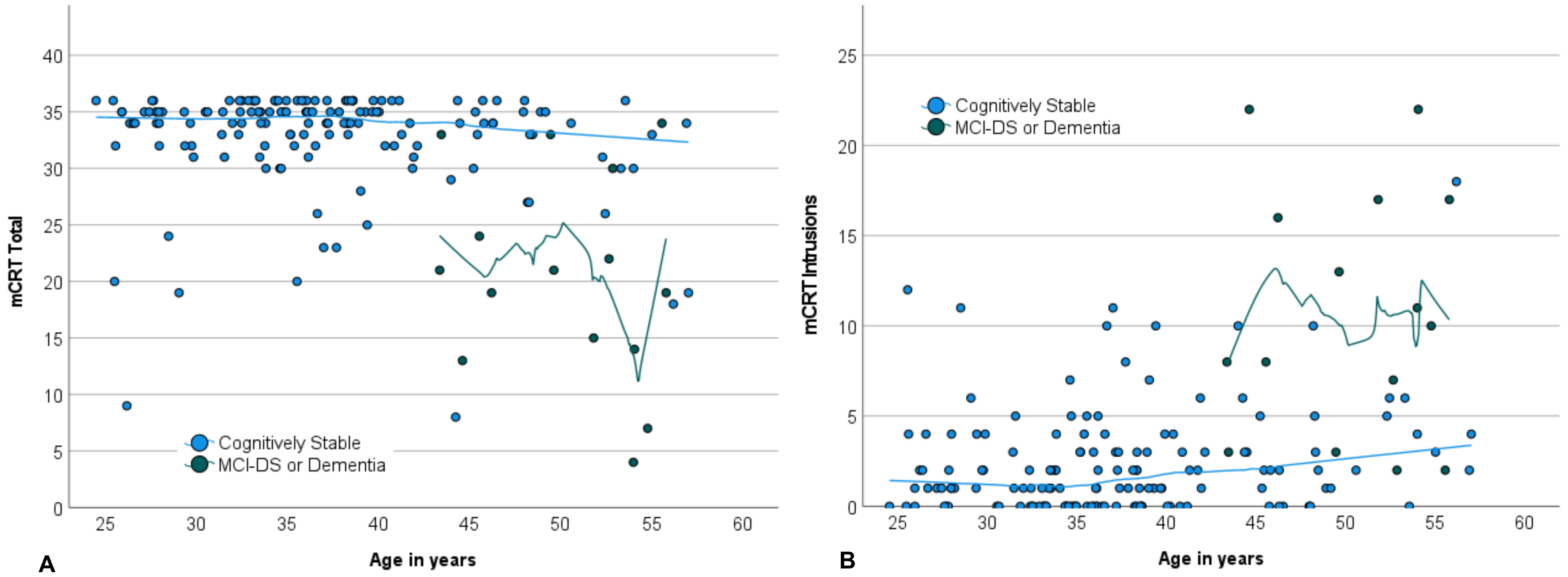

Supplemental Figure e3. Association between age in years and modified Cued Recall Total (mCRT) Total (A) and Intrusion (B) scores by clinical status of mild cognitive impairment-Down syndrome (MCI-DS) and dementia.
